# Supplementary figures and images for: Additional feedforward mechanism of Parkin activation via binding of phospho-UBL and RING0 in trans
Source: eLife. 2024 Sep 2;13:RP96699. doi: 10.7554/eLife.96699 (PMC11368401; doi:10.7554/eLife.96699)

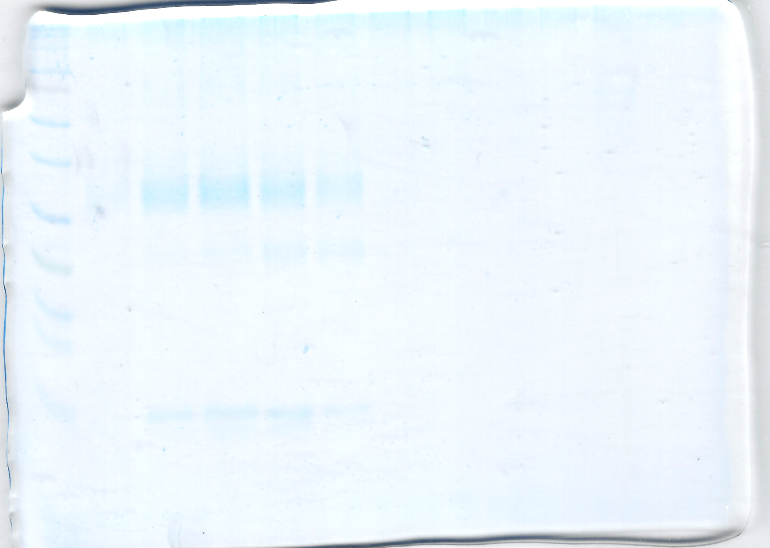

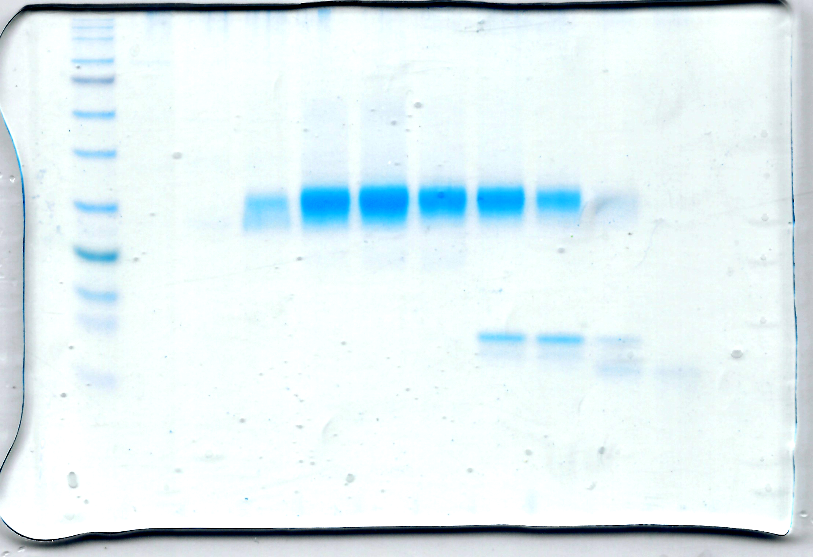

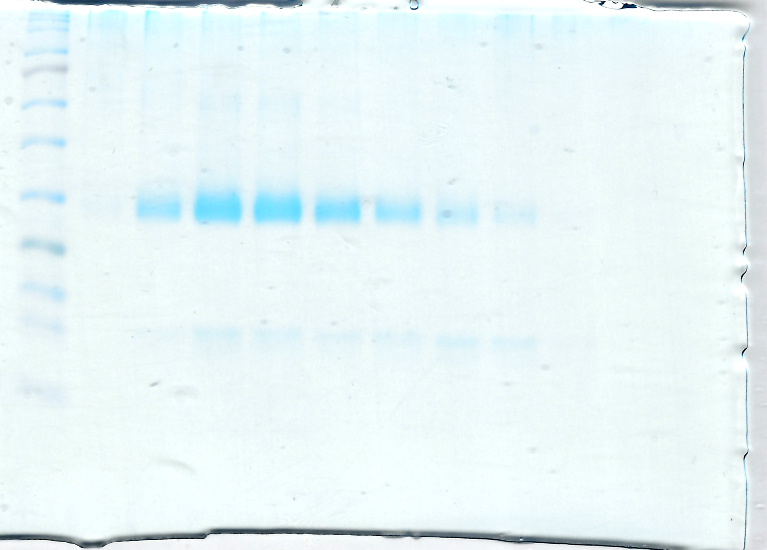

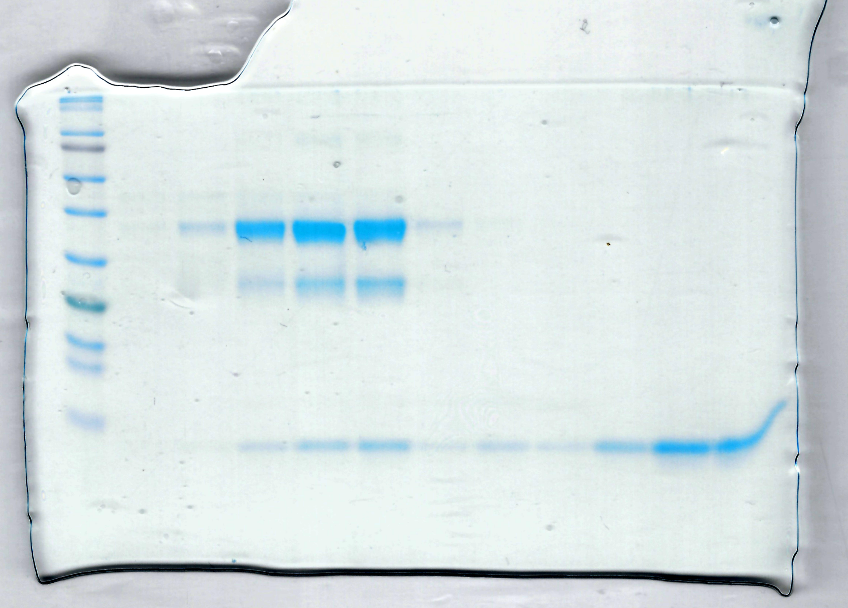


Figure 1D

Figure 1D

Figure 1C

Figure 1C

Supplement: Figure 1—source data 1. [file elife-96699-fig1-data1.docx]

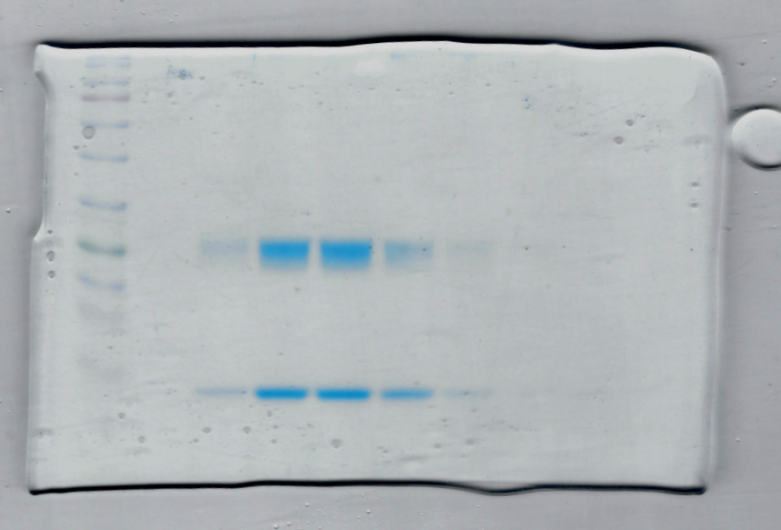

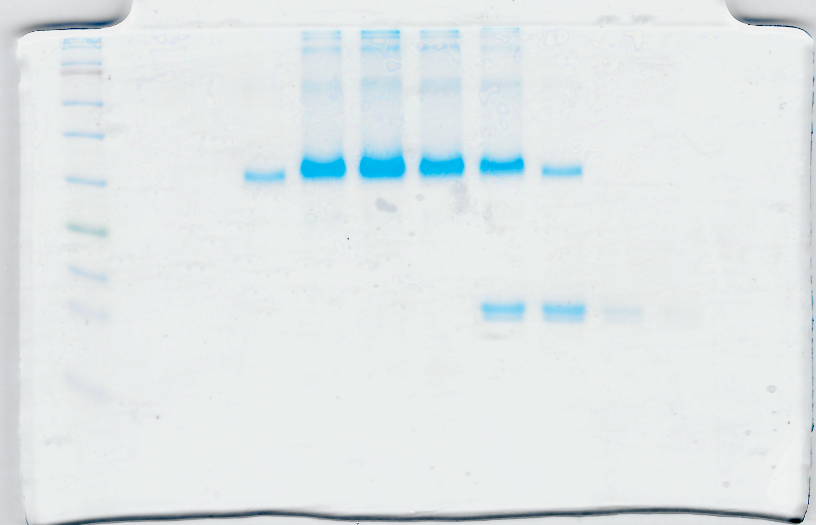

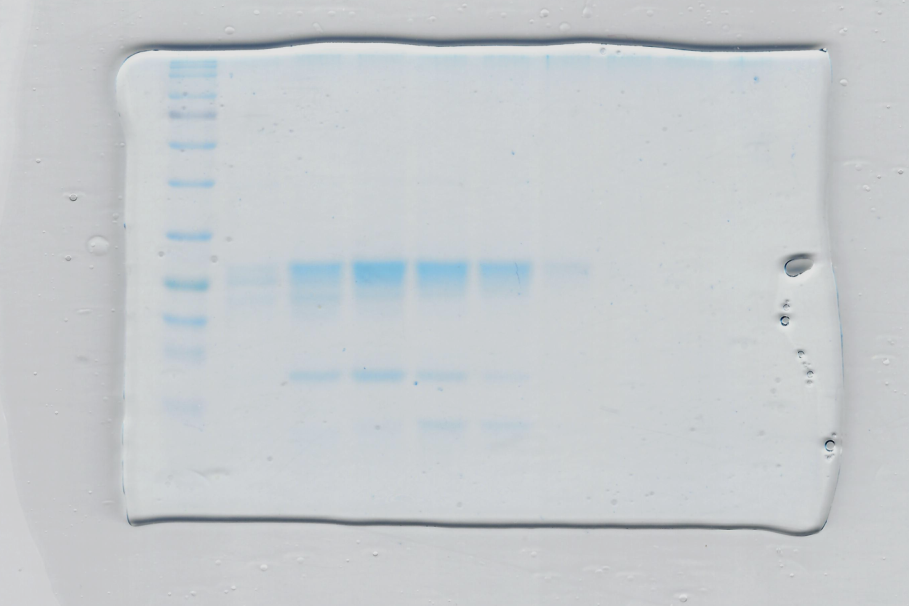

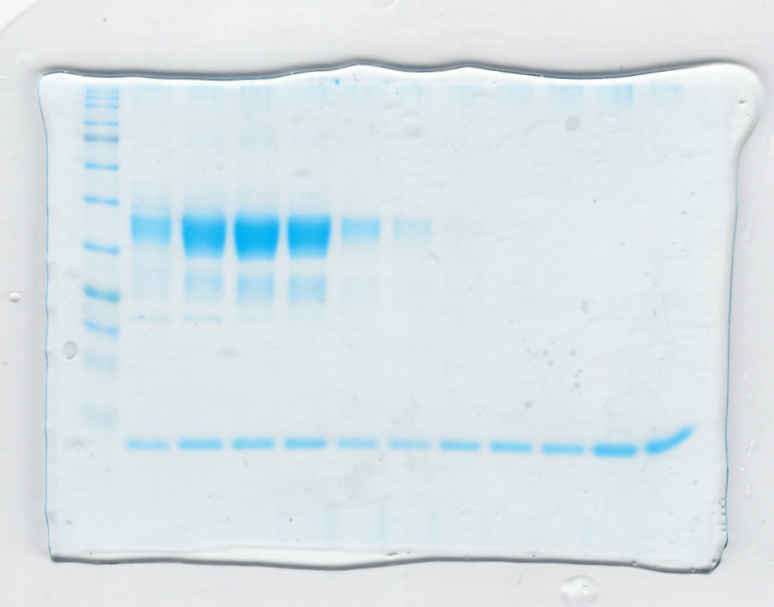


Figure 2B

Figure 2B

Figure 2A

Figure 2A

Supplement: Figure 2—source data 1. [file elife-96699-fig2-data1.docx]

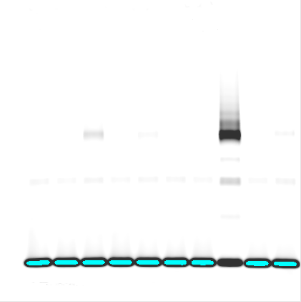

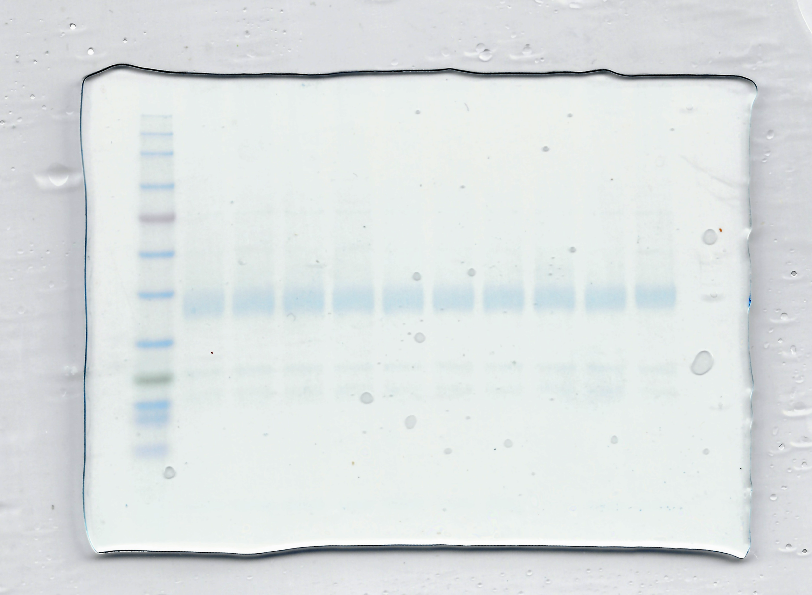

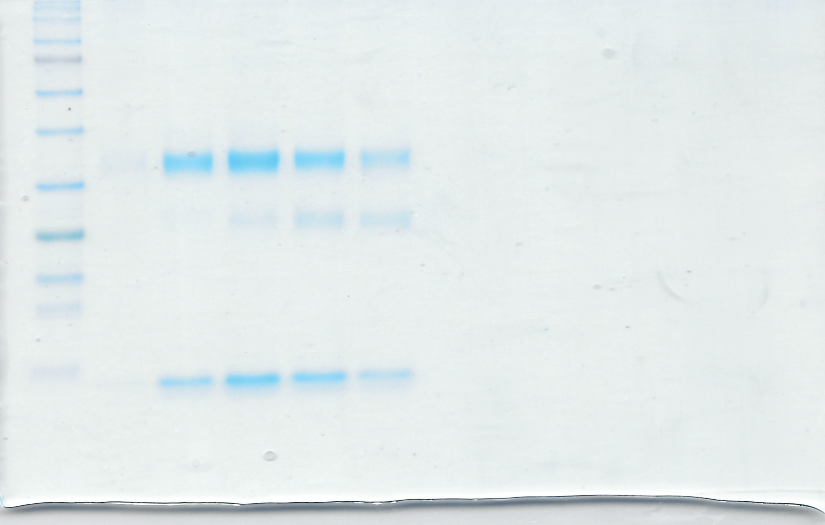

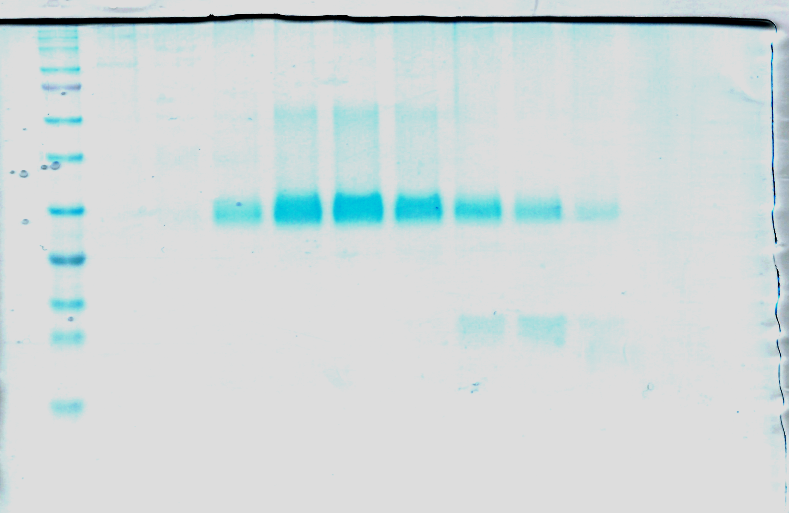

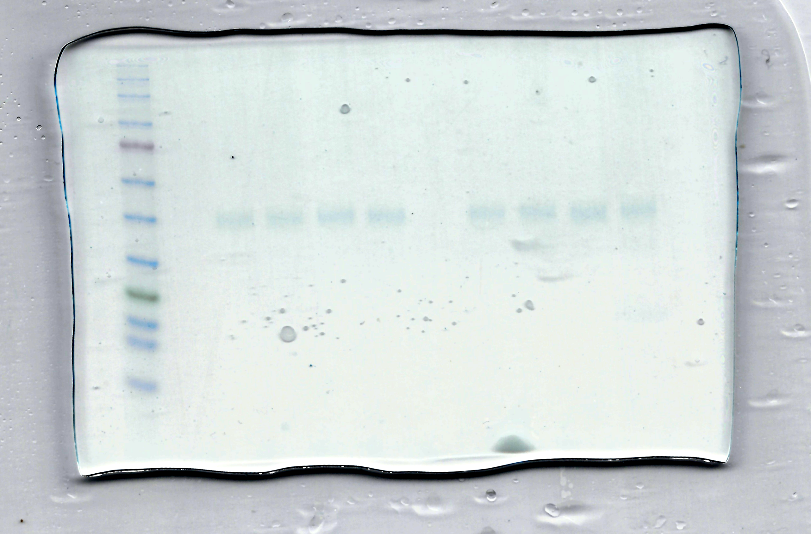

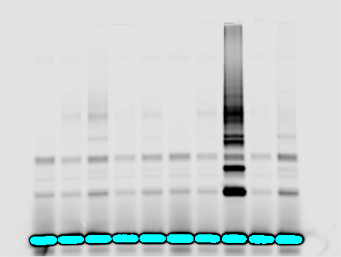


Figure 3A

Figure 3A

Figure 3B

Figure 3B

Figure 3B

Figure 3B

Supplement: Figure 3—source data 1. [file elife-96699-fig3-data1.docx]

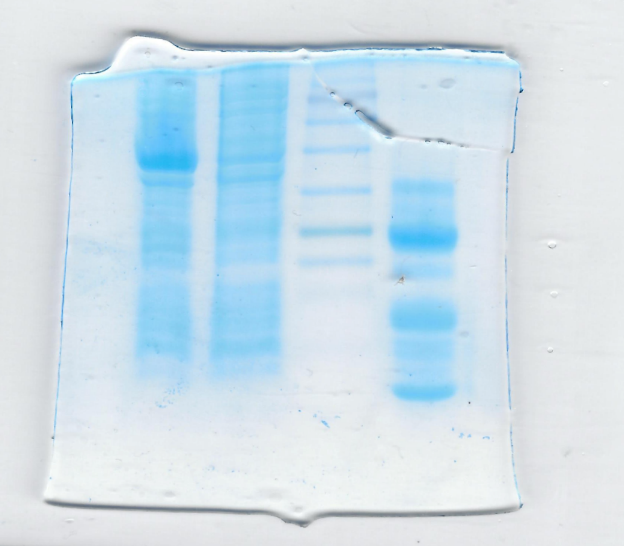

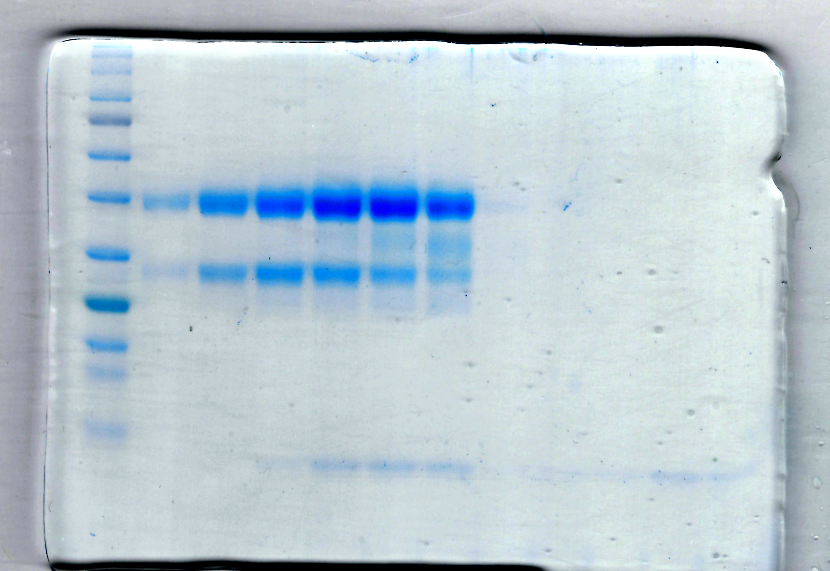

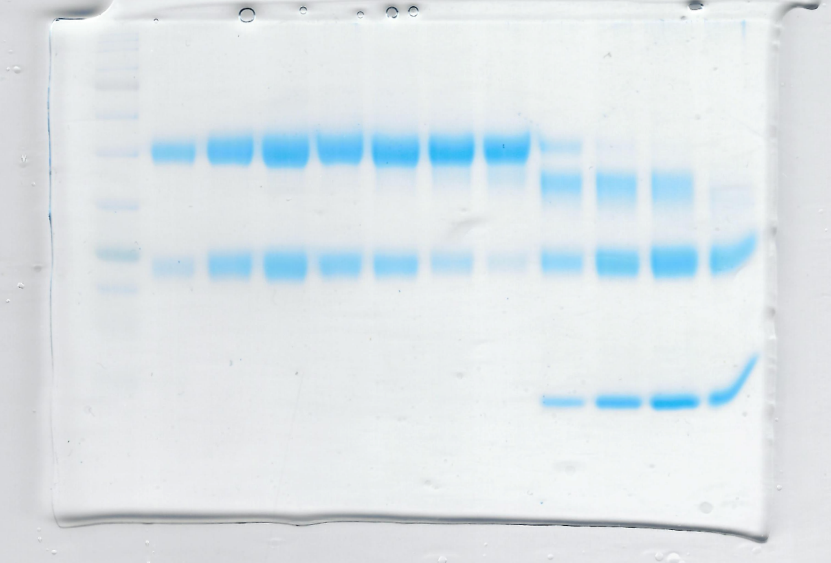

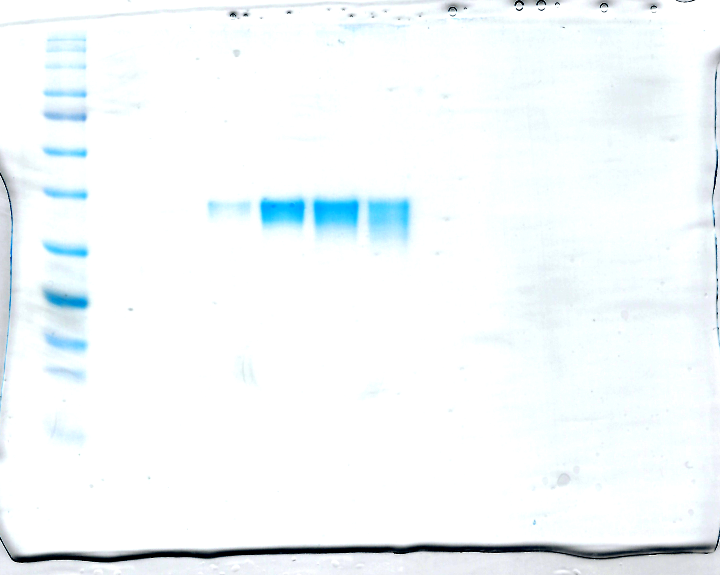

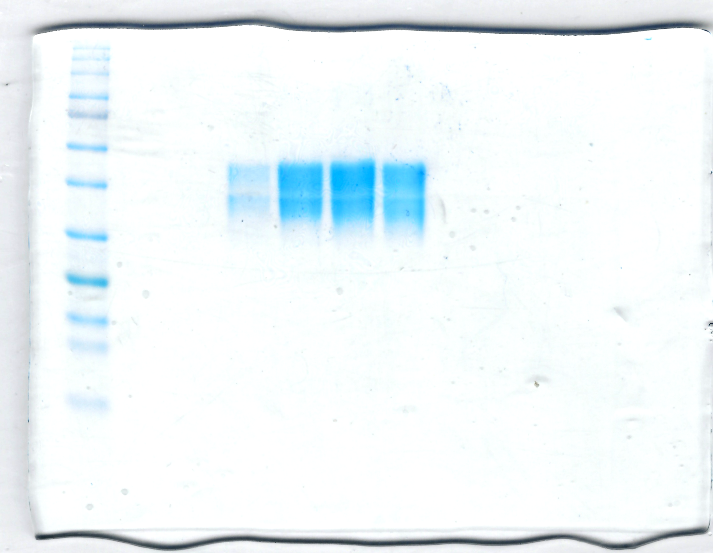

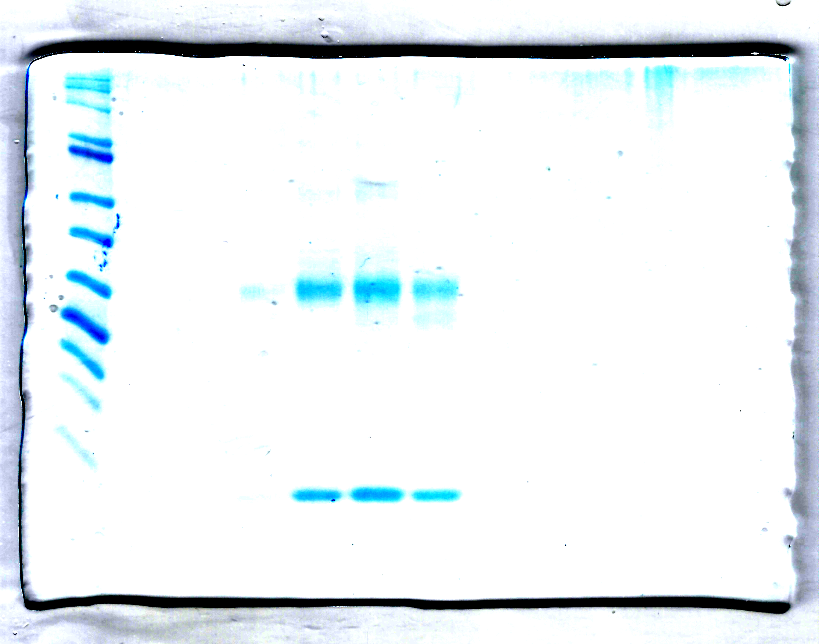


Figure 4C

Figure 4C

Not used in this study

Figure 4B

Figure 4B

Figure 4A

Figure 4A

Supplement: Figure 4—source data 1. [file elife-96699-fig4-data1.docx]

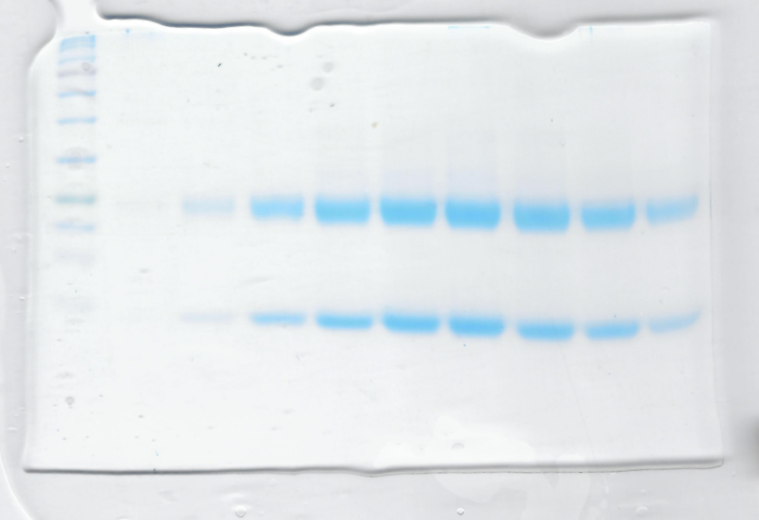


Figure 4-figure supplement 1B

Supplement: Figure 4—figure supplement 1—source data 1. [file elife-96699-fig4-figsupp1-data1.docx]

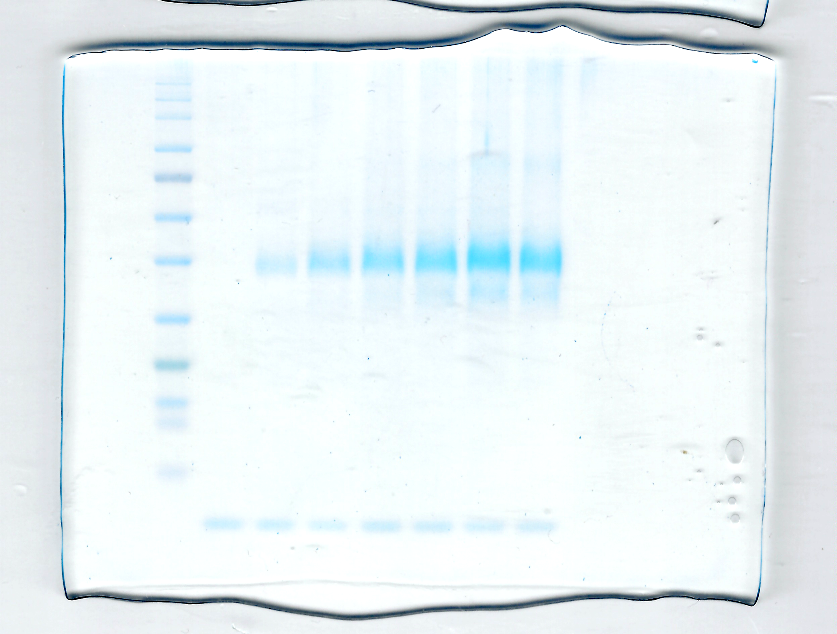



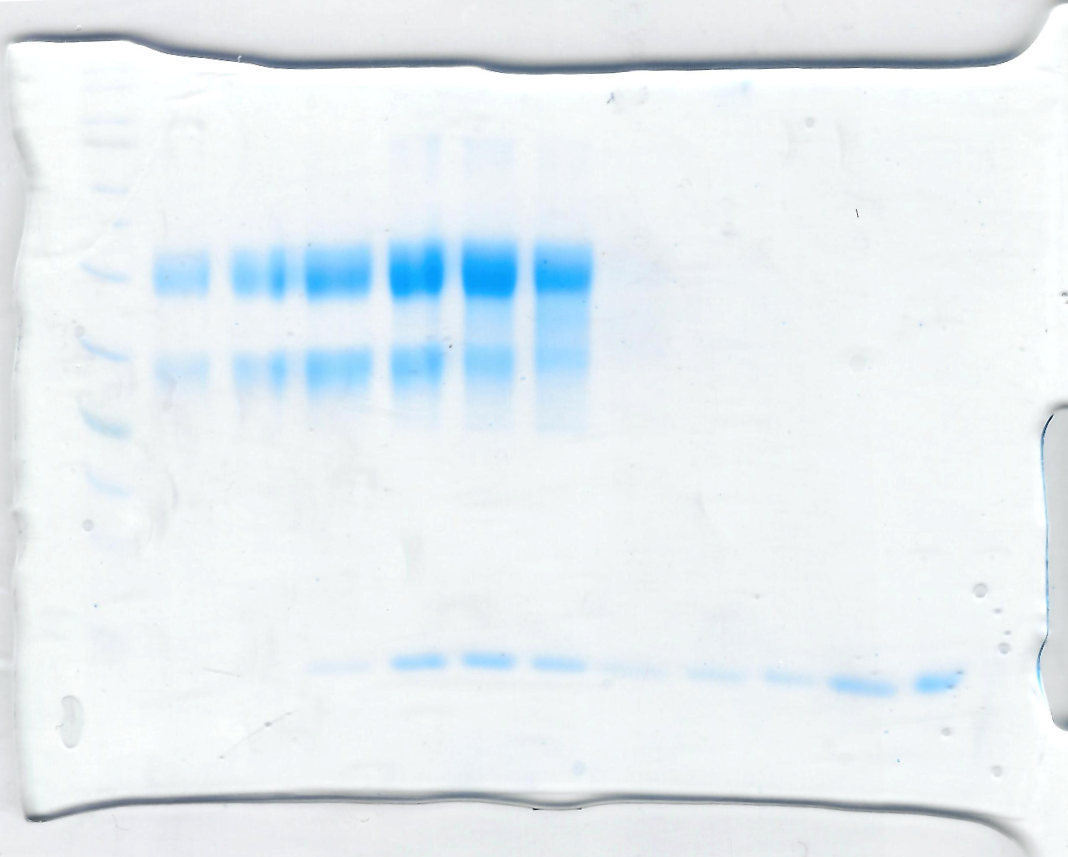

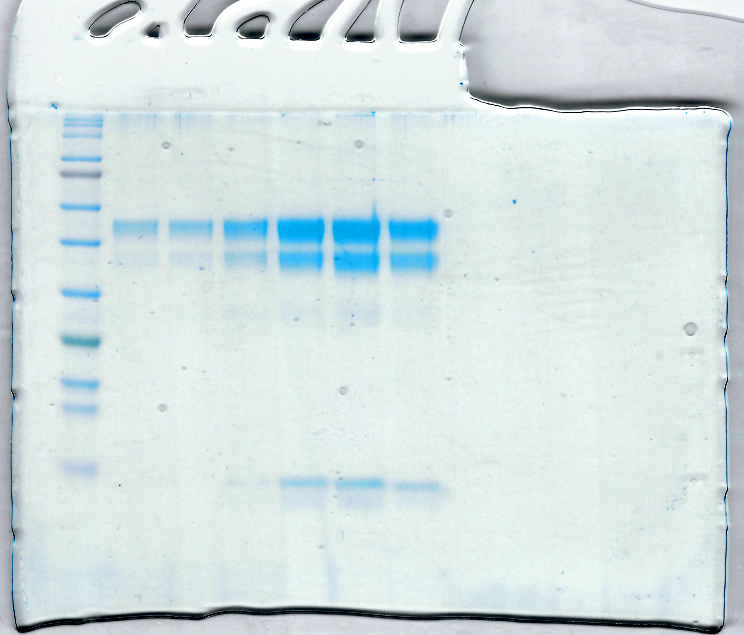

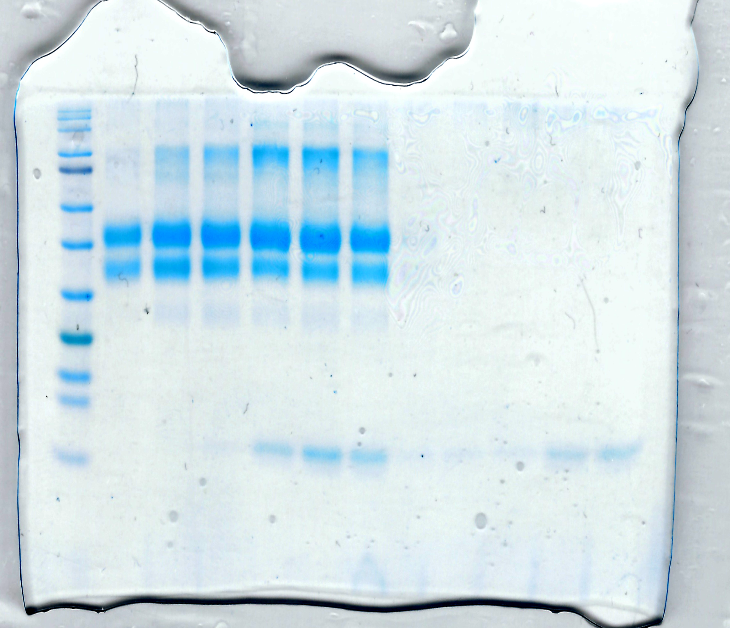


Figure 5E

Figure 5E

Figure 5A

Figure 5B

Figure 5B

Supplement: Figure 5—source data 1. [file elife-96699-fig5-data1.docx]

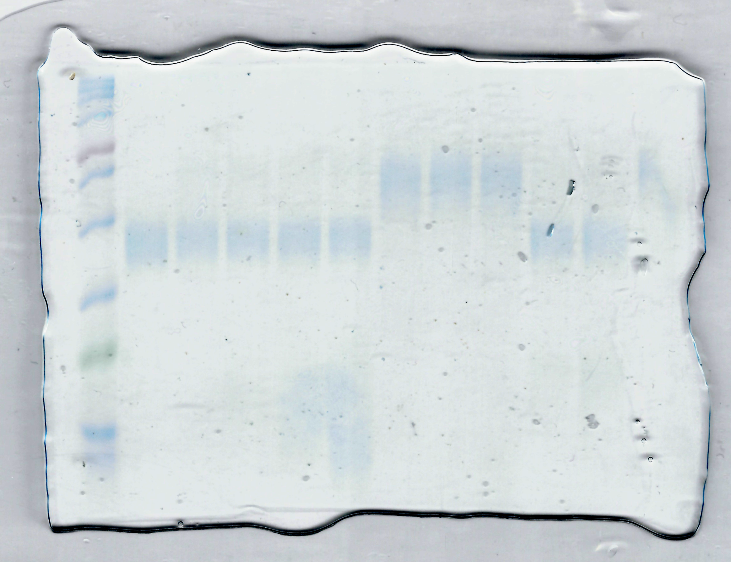


Figure 5-figure supplement 2D

Dot used in this study

Supplement: Figure 5—figure supplement 2—source data 1. [file elife-96699-fig5-figsupp2-data1.docx]

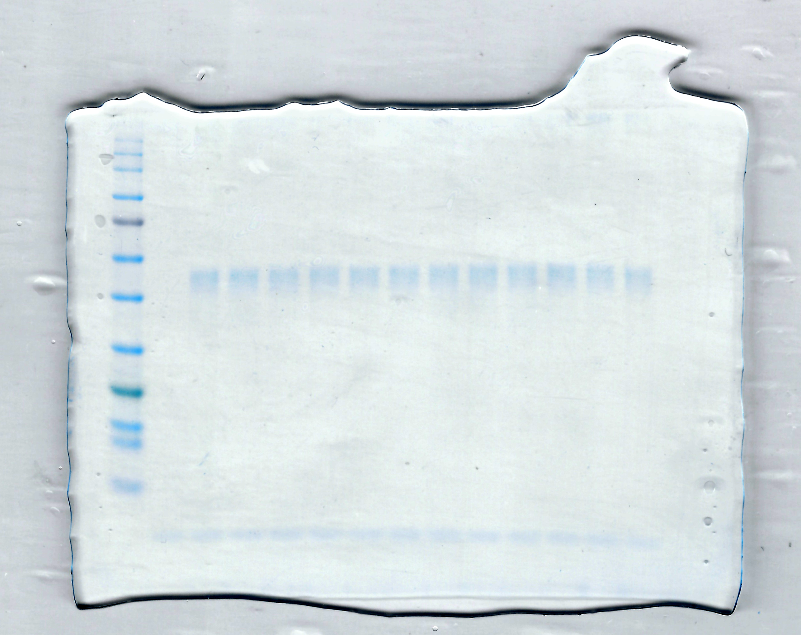



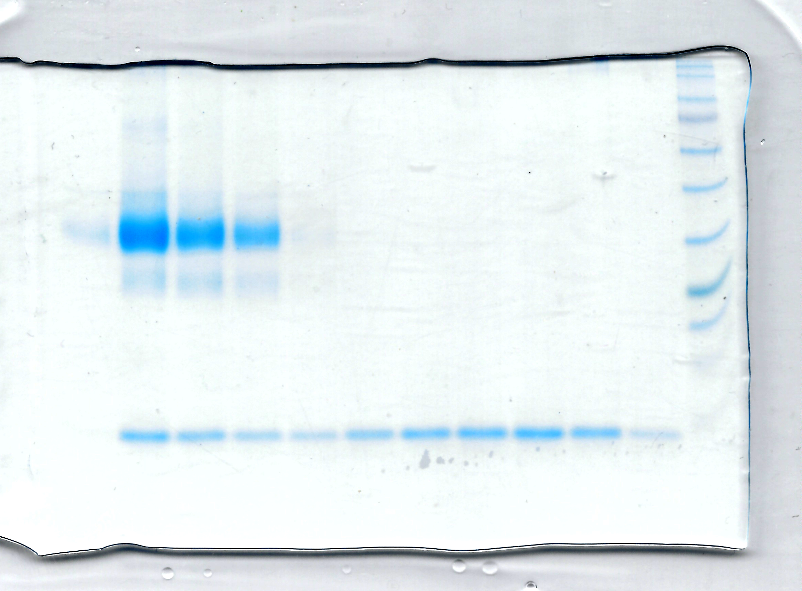

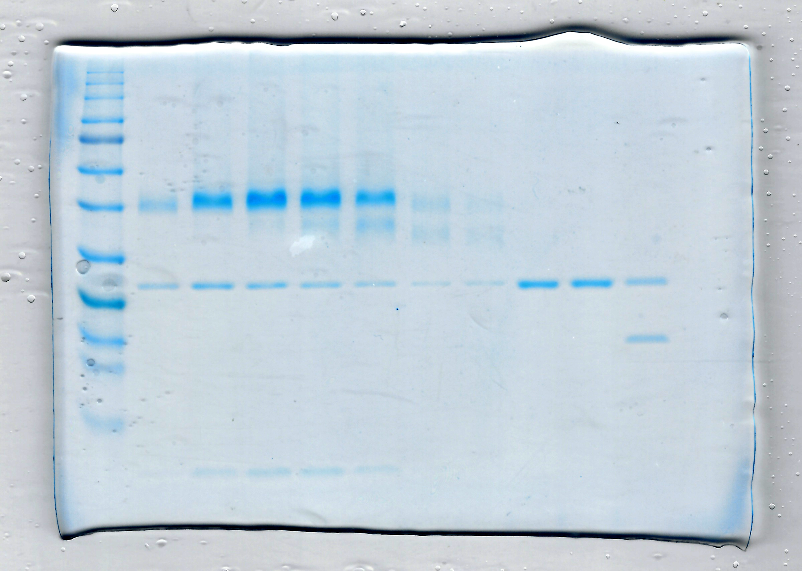

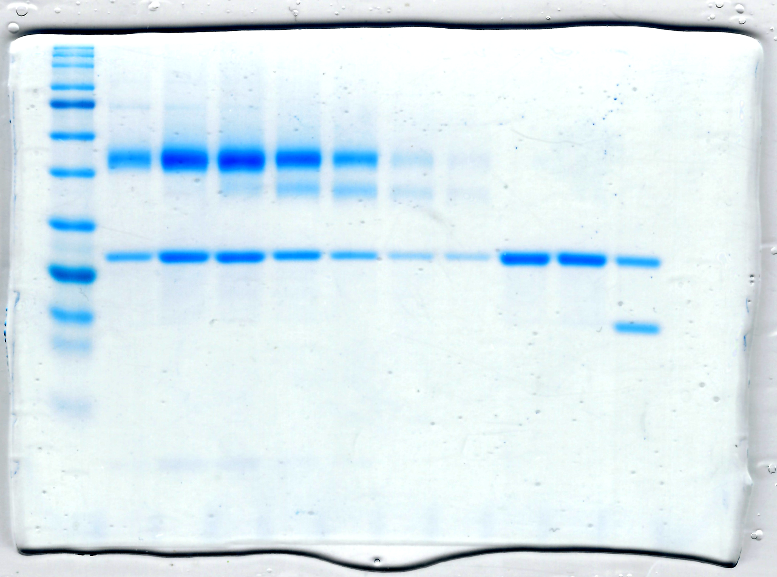


Figure 7C

Figure 7C

Figure 7B

Figure 7A

Figure 7A

Supplement: Figure 7—source data 1. [file elife-96699-fig7-data1.docx]

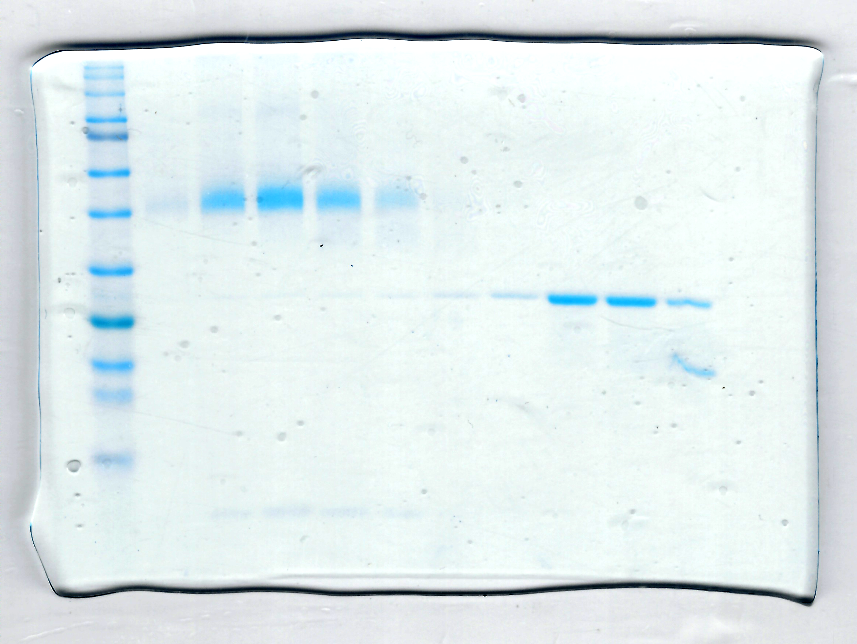

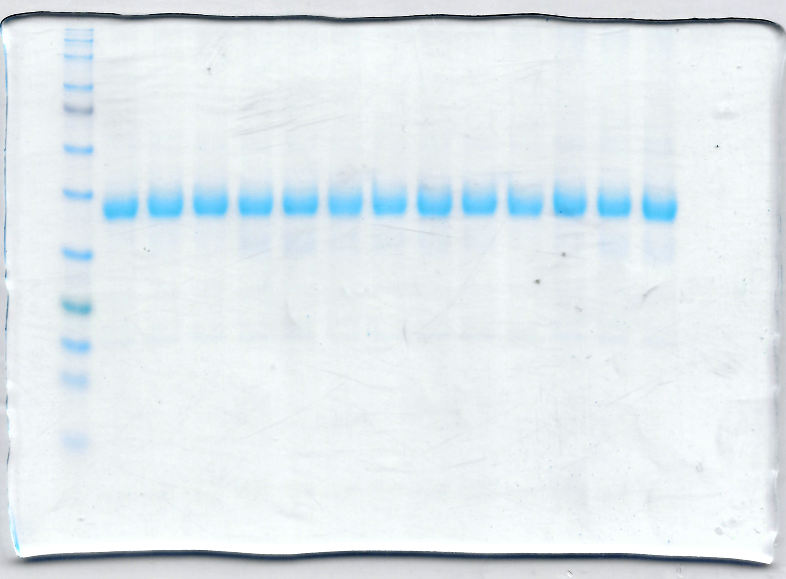

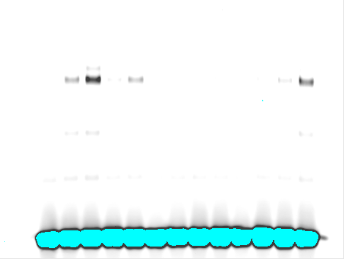

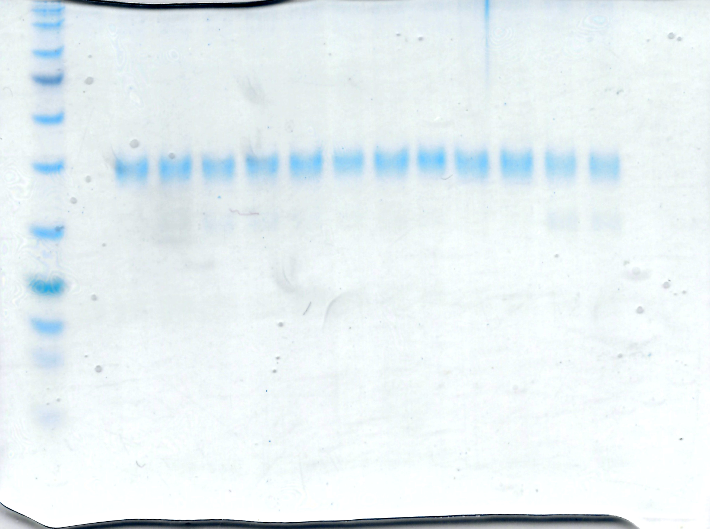

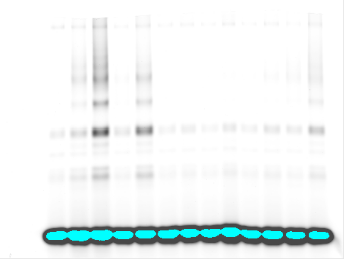


Figure 9F

Figure 9E

Figure 9E

Figure 9E

Figure 9E

Supplement: Figure 9—source data 1. [file elife-96699-fig9-data1.docx]

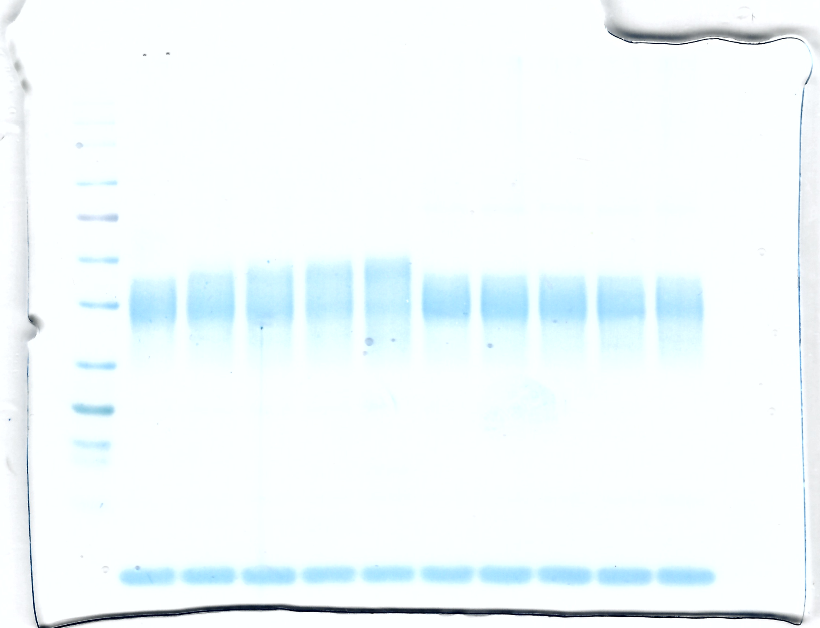

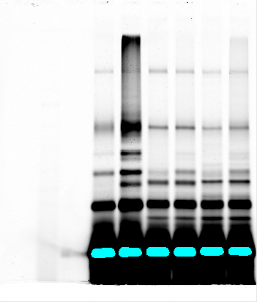

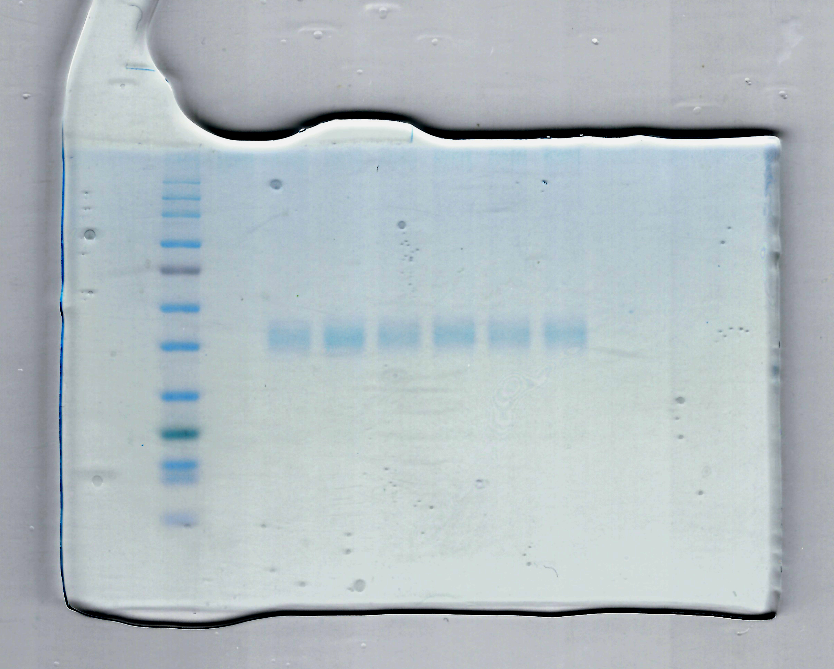


Figure 9-figure supplement 2B

Figure 9-figure supplement 2C

Figure 9-figure supplement 2C

Supplement: Figure 9—figure supplement 2—source data 1. [file elife-96699-fig9-figsupp2-data1.docx]
