# Supplementary material for: Additional feedforward mechanism of Parkin activation via binding of phospho-UBL and RING0 in trans
Source: eLife. 2024 Sep 2;13:RP96699. doi: 10.7554/eLife.96699 (PMC11368401; doi:10.7554/eLife.96699)
Supplement: Figure 1—figure supplement 2—source data 1. [file elife-96699-fig1-figsupp2-data1.docx]

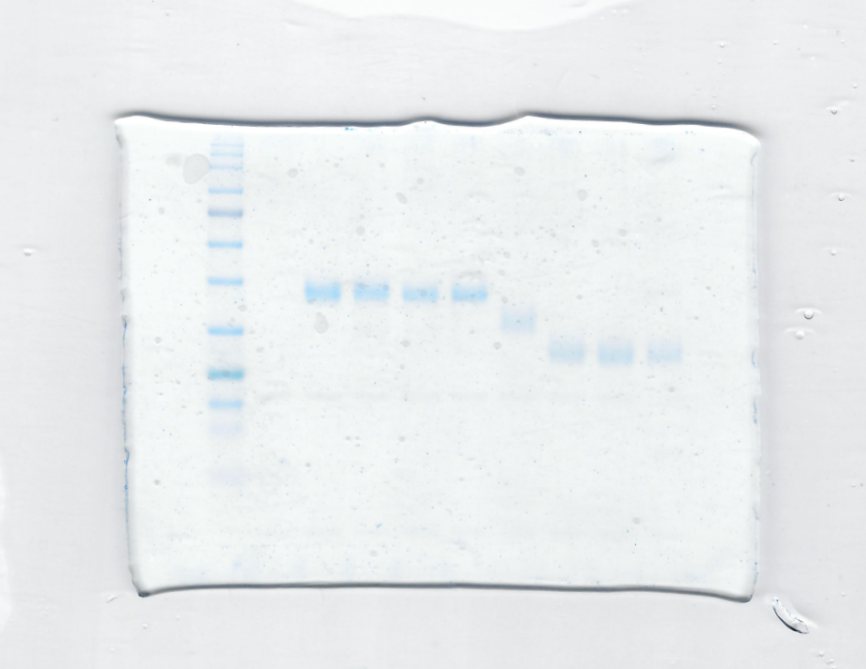

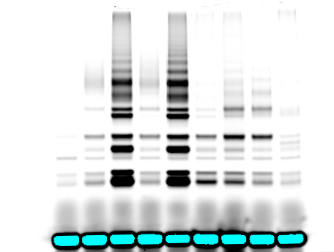


Not used in this study

Not used in this study

Figure 1-figure supplement 2

Figure 1-figure supplement 2
