# Supplementary material for: Additional feedforward mechanism of Parkin activation via binding of phospho-UBL and RING0 in trans
Source: eLife. 2024 Sep 2;13:RP96699. doi: 10.7554/eLife.96699 (PMC11368401; doi:10.7554/eLife.96699)
Supplement: Figure 8—source data 1. [file elife-96699-fig8-data1.docx]

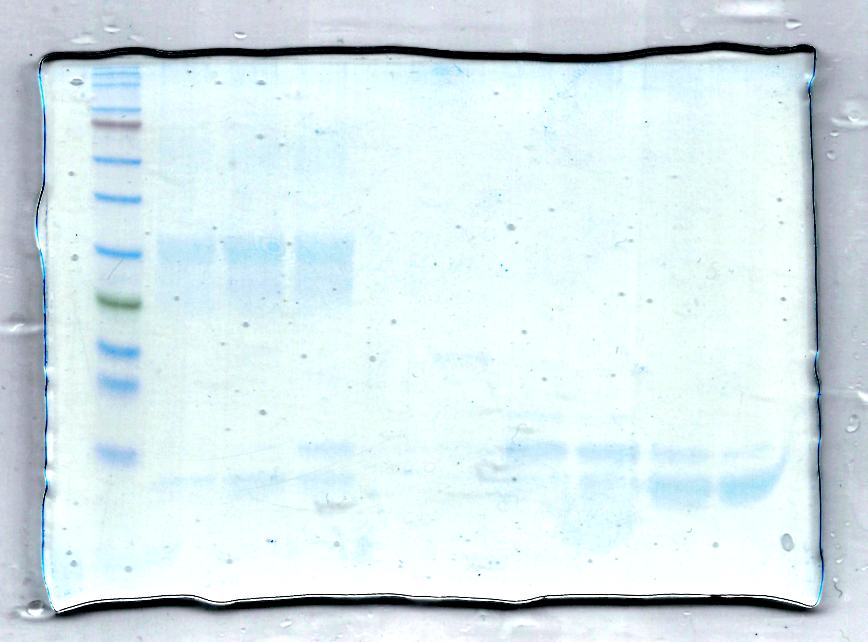

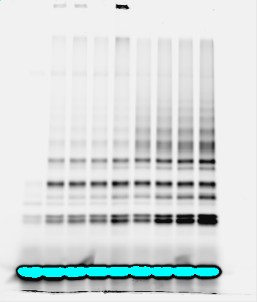

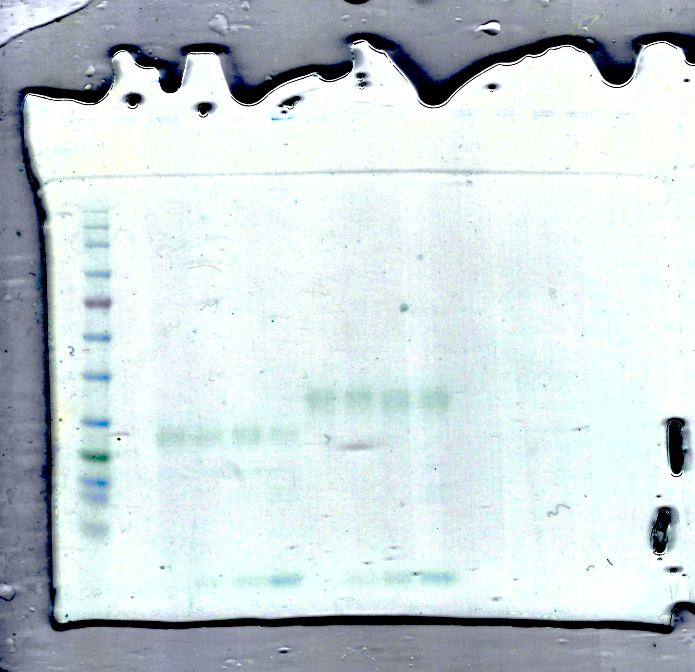


Figure 8D

Figure 8D

Figure 8B


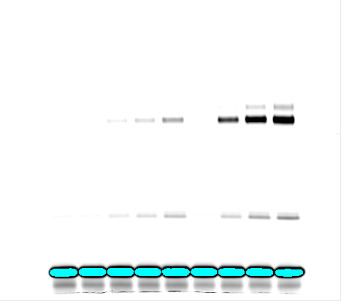

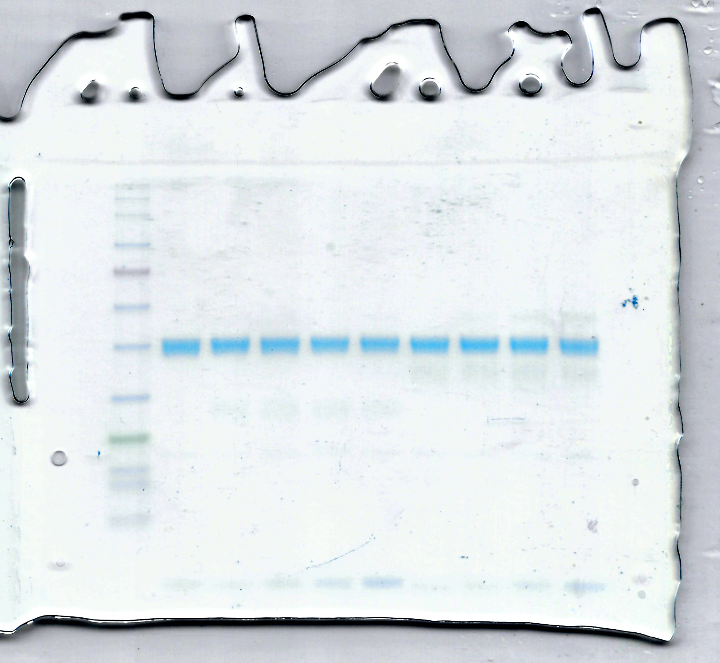


Figure 8D

Figure 8D


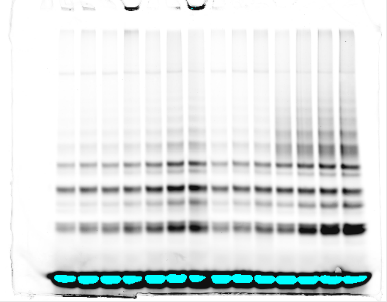

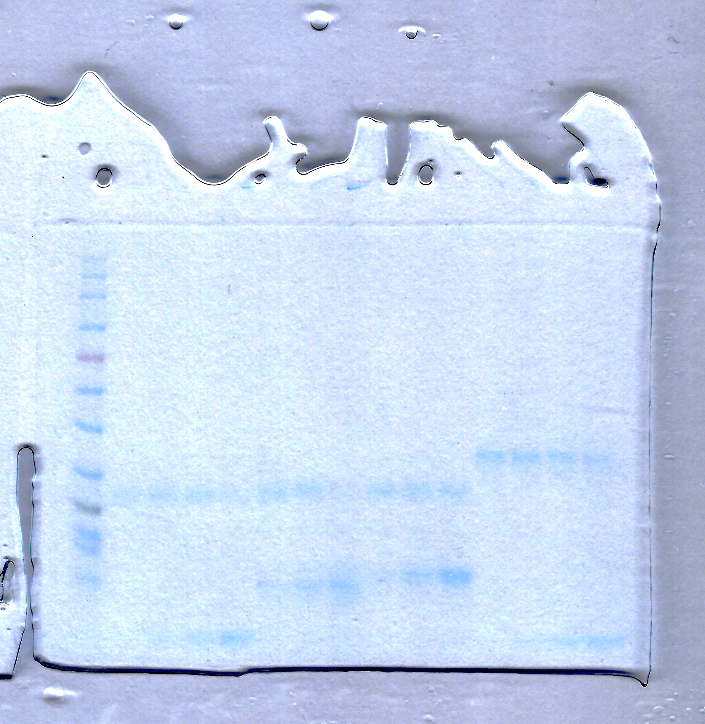

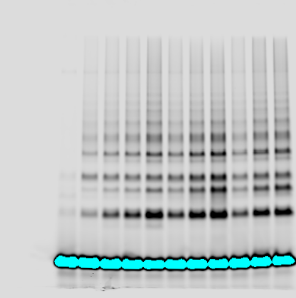

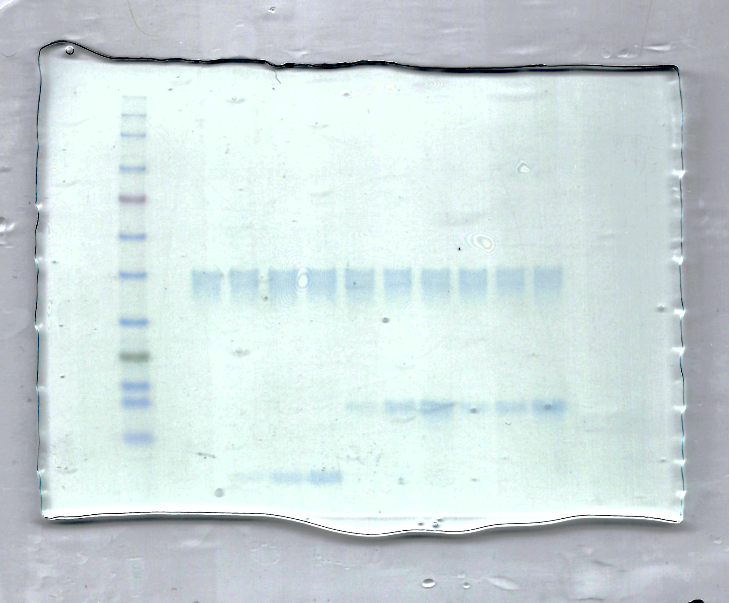

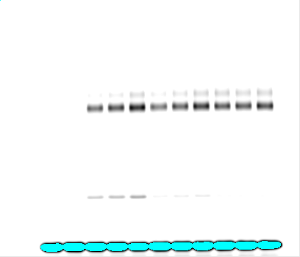

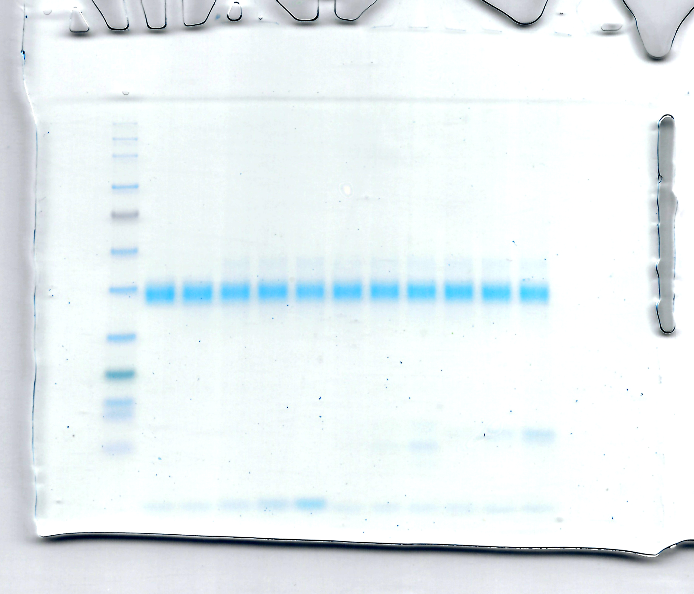


Figure 8F

Figure 8F

Figure 8E

Figure 8E

Figure 8E

Figure 8E


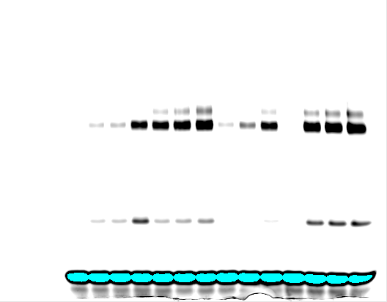

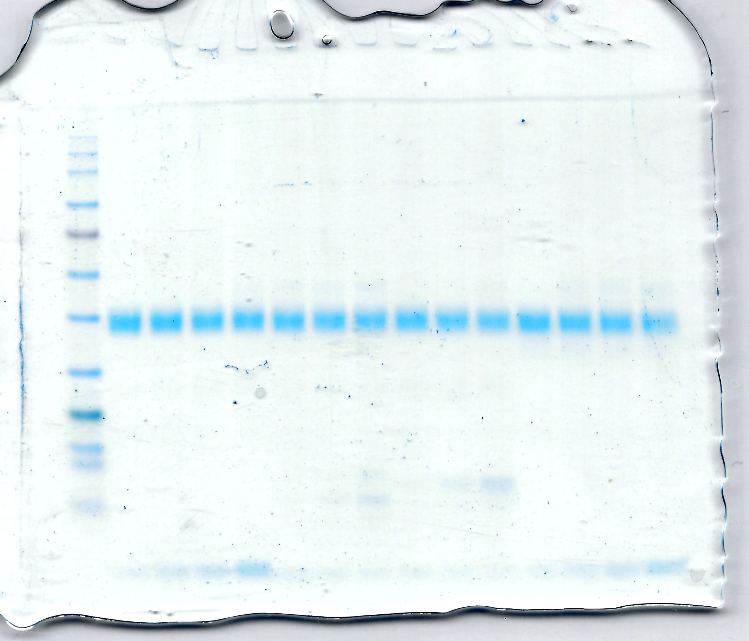


Figure 8F

Figure 8F
