# Supplementary material for: Additional feedforward mechanism of Parkin activation via binding of phospho-UBL and RING0 in trans
Source: eLife. 2024 Sep 2;13:RP96699. doi: 10.7554/eLife.96699 (PMC11368401; doi:10.7554/eLife.96699)
Supplement: Figure 8—figure supplement 1—source data 1. [file elife-96699-fig8-figsupp1-data1.docx]

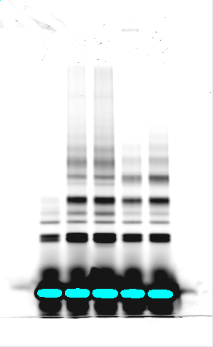

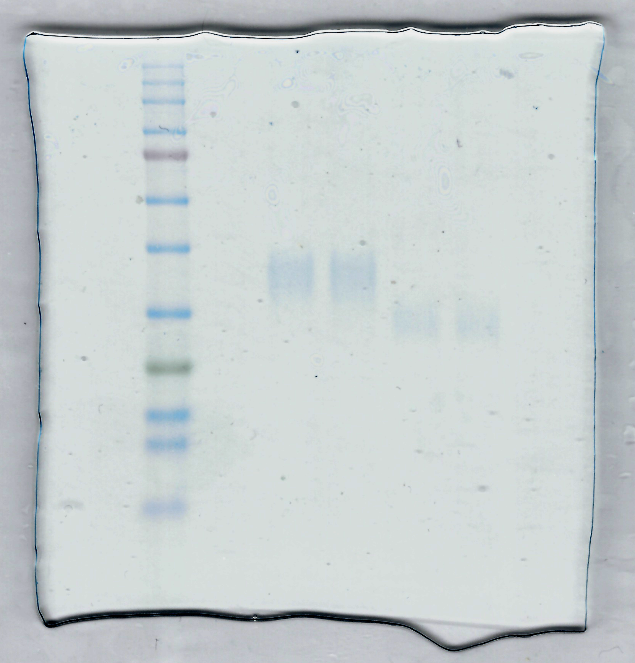

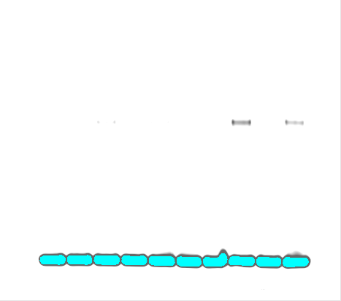

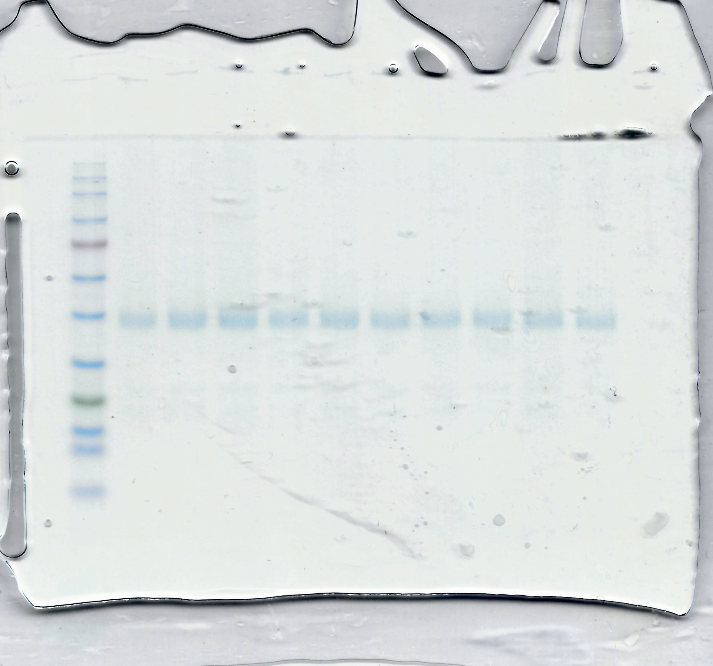


Not used in this study

Figure 8-figure supplement 1C

Figure 8-figure supplement 1C

Figure 8-figure supplement 1C

Figure 8-figure supplement 1C

Not used in this study
